# The Effect of Daylight-Saving Time on Percutaneous Coronary Intervention Outcomes in Acute Coronary Syndrome Patients—Data from the Polish National Registry of Percutaneous Coronary Interventions (ORPKI) in the Years 2014–2022

**DOI:** 10.3390/jcdd10090375

**Published:** 2023-09-01

**Authors:** Karol Kaziród-Wolski, Aleksandra Piotrowska, Janusz Sielski, Patrycja Zając, Krzysztof P. Malinowski, Michał Zabojszcz, Kamil Pytlak, Magdalena Wolska, Agnieszka Kołodziej, Mamas A. Mamas, Paulina Mizera, Zbigniew Siudak

**Affiliations:** 1 Collegium Medicum, Jan Kochanowski University in Kielce, 25-317 Kielce, Poland; karol.kazirod-wolski@ujk.edu.pl (K.K.-W.); alemarpiotrowska@gmail.com (A.P.); jsielski7@interia.pl (J.S.); michal.zabojszcz@gmail.com (M.Z.); pytlak.kam@gmail.com (K.P.); agusiat3@o2.pl (A.K.); paulinapmizera20@gmail.com (P.M.); 2 Intensive Cardiac Care Unit, Świętokrzyskie Cardiology Center, 25-736 Kielce, Poland; 3 The Rheumatology Department, The Province Hospital in Końskie, 26-200 Końskie, Poland; patrycjazajac1716@gmail.com; 4 Department of Bioinformatics and Telemedicine, Jagiellonian University Medical College, 30-688 Kraków, Poland; krzysztof.piotr.malinowski@gmail.com; 5 Outpatient Treatment Facility “CenterMed”, 25-314 Kielce, Poland; mag.wolska@poczta.onet.pl; 6 Keele Cardiac Research Group, Keele University, Keele ST5 5BG, UK; mamasmamas1@yahoo.co.uk

**Keywords:** acute coronary syndromes, mortality, myocardial infarction, unstable angina, winter/summer time transition

## Abstract

Introduction: Many factors related to the switch to summer/winter time interfere with biological rhythms. Objectives: This study aimed to analyze the impact of time change on clinical outcomes of patients with acute coronary syndromes (ACS) undergoing percutaneous coronary intervention (PCI). Patients and methods: Electronic data of 874,031 patients with ACS who underwent invasive procedures were collected from the Polish National Register of Interventional Cardiology Procedures (ORPKI) between 2014 and 2021. We determined the number of patients undergoing PCI and periprocedural mortality during the day of spring or autumn time change and within the first 3 and 7 days after the time change. Results: We demonstrated the impact of time changes on the periprocedural mortality of ACS patients within 1 day and the period of 3 and 7 days from the time change. We observed that the occurrence of all ACS and NSTEMI on the first day was lower for both time changes and higher in the case of UA and spring time change. The autumn time change significantly reduced the occurrence of all types of ACS. A significant decrease in the number of invasive procedures was found after autumn transition in the period from the first day to 7 days for ACS, NSTEMI, and UA. Conclusions: The occurrence of ACS and the number of invasive procedures were lower for both changes over time. Autumn time change is associated with increased periprocedural mortality in ACS and a less frequent occurrence of UA and NSTEMI within 7 days.

## 1. Introduction

Biological rhythms are natural cycles that, depending on the length of the cycle, can be divided into circadian (with a period close to 24 h), ultradial (with a period of less than 24 h), and infradial (with a period longer than 24 h, e.g., monthly and yearly) [1]. The circadian rhythms and their impact on human health have been extensively studied. Previous studies have demonstrated that endogenous clock dysregulation is associated with an increase in the risk of cardiovascular and metabolic diseases and aging, as well as a higher risk of cancer [2,3].

The cardiovascular system also exhibits circadian changes. There is a trend for AMI to occur in the morning [4]. The occurrence of cardiovascular events coincides with the variability of triggering mechanisms and factors, such as elevated blood pressure, sympathetic activity, vasoconstrictor hormones, increased viscosity, fibrinogen, platelet aggregation, and decreased fibrinolytic activity [1]. There are many factors that can interfere with biological rhythms, including the exposure to light in the evening and at night, intercontinental travel with a rapid change in time zones, irregular times of activity and sleep, and shift work. Torquati et al. conducted a risk analysis of cardiovascular events in a large group of patients working in a shift work system. They estimated that the risk of death due to cardiovascular disease (CVD) and coronary heart disease (CHD) was 20% higher in shift workers compared to daytime workers [5].

The disruption of the endogenous circadian rhythm can also be influenced by the change in time related to the switch to summer/winter time. Germany was the first country in the world to introduce a time change in 1916, which was due to an implemented approach to save energy needed to illuminate factories. Some recent studies have suggested that such change in time (mainly to the summer) is associated with an increased risk of AMI [1,6]; however, others failed to demonstrate such a relationship [7]. In order to better understand this phenomenon, we analyzed the impact of time change on in-hospital clinical outcomes of patients with acute coronary syndromes undergoing percutaneous coronary intervention (PCI) included in the Polish National Register of Interventional Cardiology Procedures (ORPKI).

## 2. Patients and Methods

### 2.1. Study Cohort

The study participants were included in the ORPKI register. The National Register of Invasive Cardiology Procedures comprises patients diagnosed with non-ST-segment-elevation myocardial infarction (NSTEMI), STEMI (ST-segment-elevation myocardial infarction), or UA (unstable angina) in whom coronary interventions (coronary angiography, PCI, or both procedures at the same time) have been performed as an emergency treatment. The ORPKI register includes patients from all over the country; moreover, 161 laboratories from Poland participate in this project.

Demographic and procedural data were collected during the patient’s admission for invasive procedures to the hospital. At that time, the doctor assisting the patient filled out a qualifying form. Follow-up data are not included in the register. Reported mortality in the Cath Lab referred to periprocedural deaths.

Electronic data were collected from patients with acute coronary syndromes who underwent invasive cardiology procedures. This study included data from patients enrolled in the register between 2014 and 2021 [8,9]. We determined the number of patients undergoing PCI following admission with ACS (STEMI, NSTEMI, or UA) during the day of autumn and spring time change. As a reference, we used the three first days after the time change, i.e., Sunday, Monday, and Tuesday. We also analyzed the entire week after the time change, i.e., from Saturday to Saturday of the following week.

The basic reference was the time of 1 day on Saturday–Sunday without any time change (defined as ordinary). However, the reference for the analysis in the perspective of the next 3 and 7 days after the change in time was the same time interval calculated from the weekend without changing the time (defined as ordinary). The weekend itself in terms of the occurrence of ACS and ASC treatment (cardiology centers work only in the ER mode from Friday to Sunday) is different than weekdays, which is why the authors decided not to include weekdays as references.

### 2.2. Statistical Analysis

Nominal variables are presented as counts and percentages, whereas continuous variables are presented as means with standard deviation as well as the median with the first and the third quartiles. A comparison of nominal variables between analyzed groups was performed using the Pearson χ^2^ test or Fisher Exact Test. Continuous variables were compared using Student’s *t*-test. For each group (defined by the daylight-saving time change), the number of ACS and their categories (STEMI, NSTEMI, and UA) were calculated and compared to each other assuming Poisson distribution and presented as rate ratios (RR) with two-sided 95% confidence intervals (95% CI). Multiple Poisson regression analyses were performed to search for the potential confounders. Each model included a term that indicated whether the change in time occurred on a specific day and was adjusted for characteristics of patients on that day—age, weight, gender, diabetes, a previous stroke, previous MI, previous PCI, previous CABG, smoking status, psoriasis, hypertension, kidney disease, and COPD—as well as to the day of the week to account for potential variation within the week. Results are presented as RR with a two-sided confidence interval (95% CI). Since comparisons were made across multiple groups, *p*-values were adjusted for multiple comparisons. The statistical analysis was performed using Stata 17.0 (StataCorp LLC, College Station, TX, USA, 2022) and JMP 16.2 (SAS Institute Inc., Cary, NC, USA, 2022).

## 3. Results

**Figure 1:**
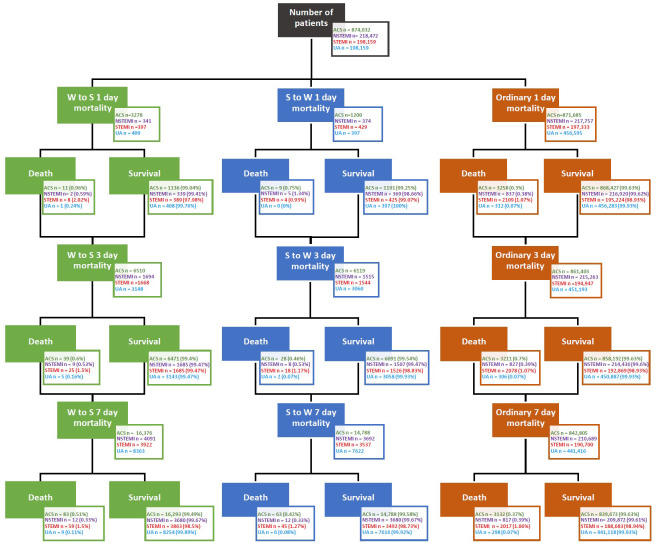
Patients’ outcome with acute coronary syndrome, non-ST-elevation myocardial infarction, ST-elevation myocardial infarction, or unstable angina after the time change. Abbreviations: ACS, acute coronary syndrome; NSTEMI, non-ST-elevation myocardial infarction; STEMI, ST-elevation myocardial infarction; S to W, Summer to Winter; UA, unstable angina; W to S, Winter to Summer.

The ORPKI register comprises a total of 874,031 percutaneous coronary interventions performed between 2014 and 2021 (2922 days). We evaluated the effect of time change by analyzing the occurrence and periprocedural mortality in ACS. Figure 1 shows the outcomes of patients undergoing invasive procedures in a specific period after daylight-saving time (DST) (1 day, 3 days, and 7 days).

**Figure 2:**
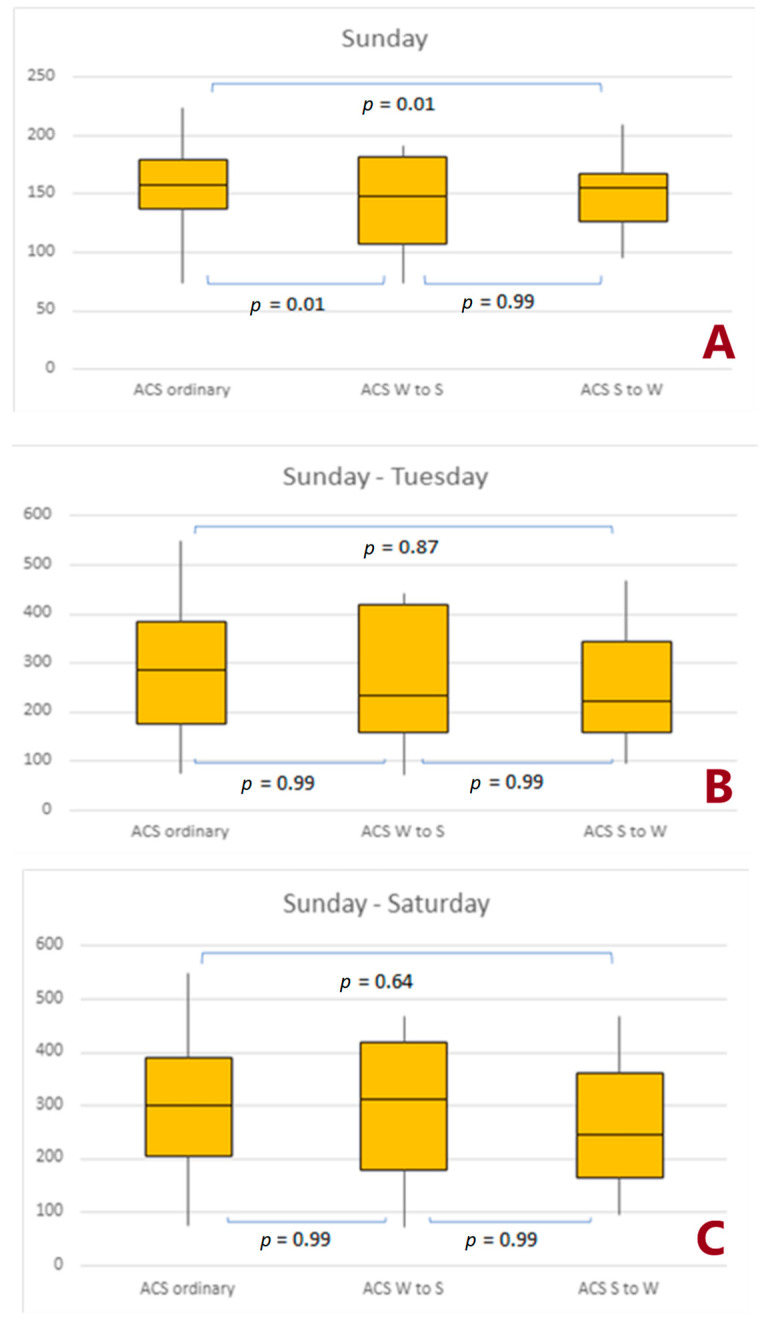
Number of acute coronary syndromes that occurred in a specific period after the time change. (**A**) 1 day effect; (**B**) 3 days effect, (**C**) 7 days effect. Abbreviations: ACS, acute coronary syndrome; S to W, Summer to Winter; W to S, Winter to Summer.

Analyzing the differences in the number of ACS occurring directly on the first day, it was found that the number of ACS was lower for both changes in time (Figure 2A). However, time change was associated with insignificant differences in ACS counts both within 3 days and 7 days after the time change (Figure 2B,C).

**Figure 3:**
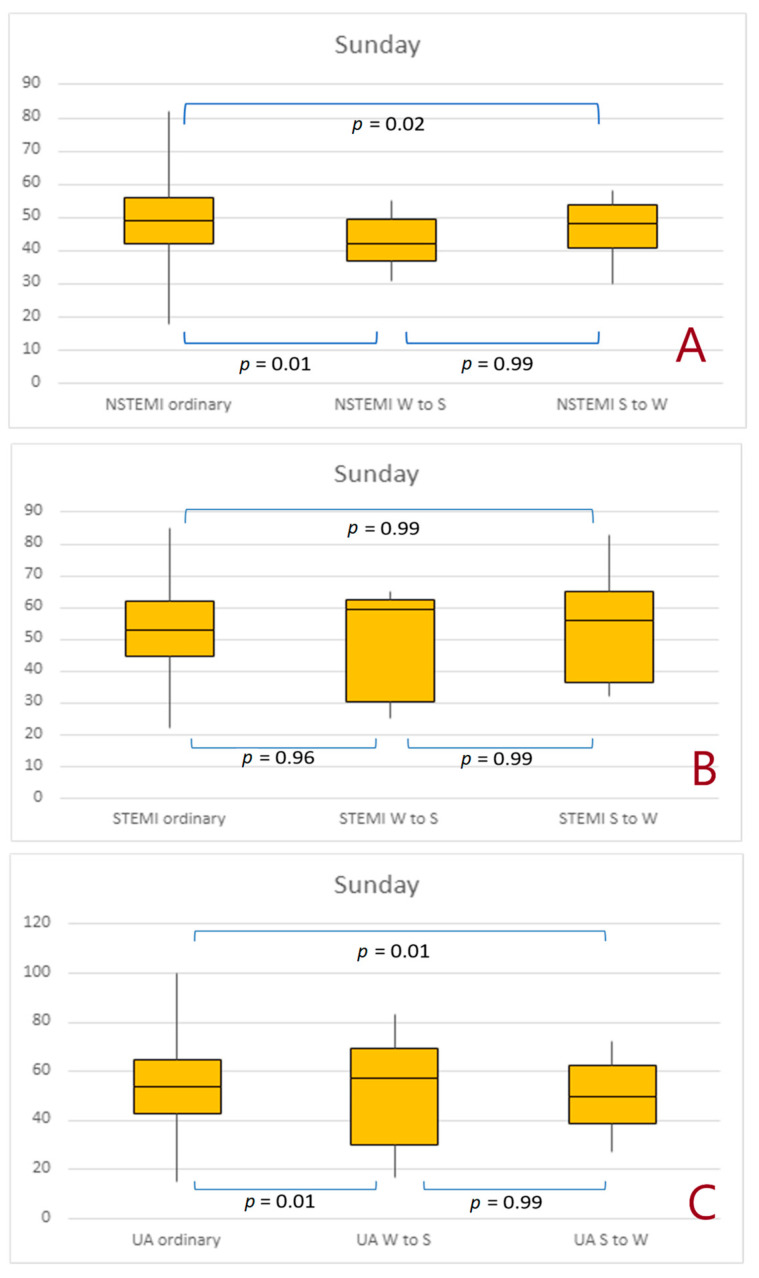
Number of non-ST-elevation myocardial infarctions, ST-elevation myocardial infarctions, and unstable angina cases that occurred on Sunday after the time change. (**A**) 1 day effect; (**B**) 3 days effect, (**C**) 7 days effect. Abbreviations: NSTEMI, non-ST-elevation myocardial infarction; STEMI, ST-elevation myocardial infarction; UA, unstable angina; S to W, Summer to Winter; W to S, Winter to Summer.

The Sunday following any time change (autumn and spring combined) was associated with a significantly lower number of NSTEMIs (Figure 3A). Moreover, we observed a higher incidence of UA on Sunday following spring time change (Figure 3C, respectively). However, such changes were not seen in STEMI (Figure 3B).

**Figure 4:**
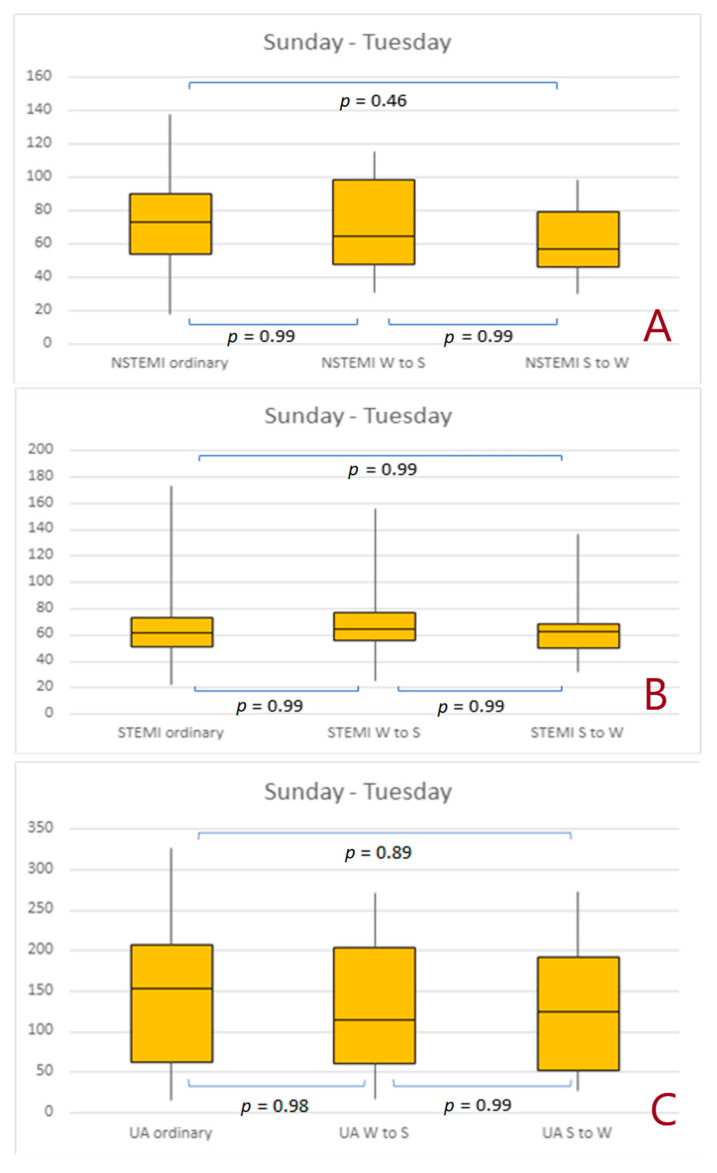
Number of non-ST-elevation myocardial infarctions, ST-elevation myocardial infarctions, and unstable angina cases that occurred between Sunday and Tuesday after the time change. (**A**) 1 day effect; (**B**) 3 days effect, (**C**) 7 days effect. Abbreviations: NSTEMI, non-ST-elevation myocardial infarction; STEMI, ST-elevation myocardial infarction; S to W, Summer to Winter; UA, unstable angina; W to S, Winter to Summer.

**Figure 5:**
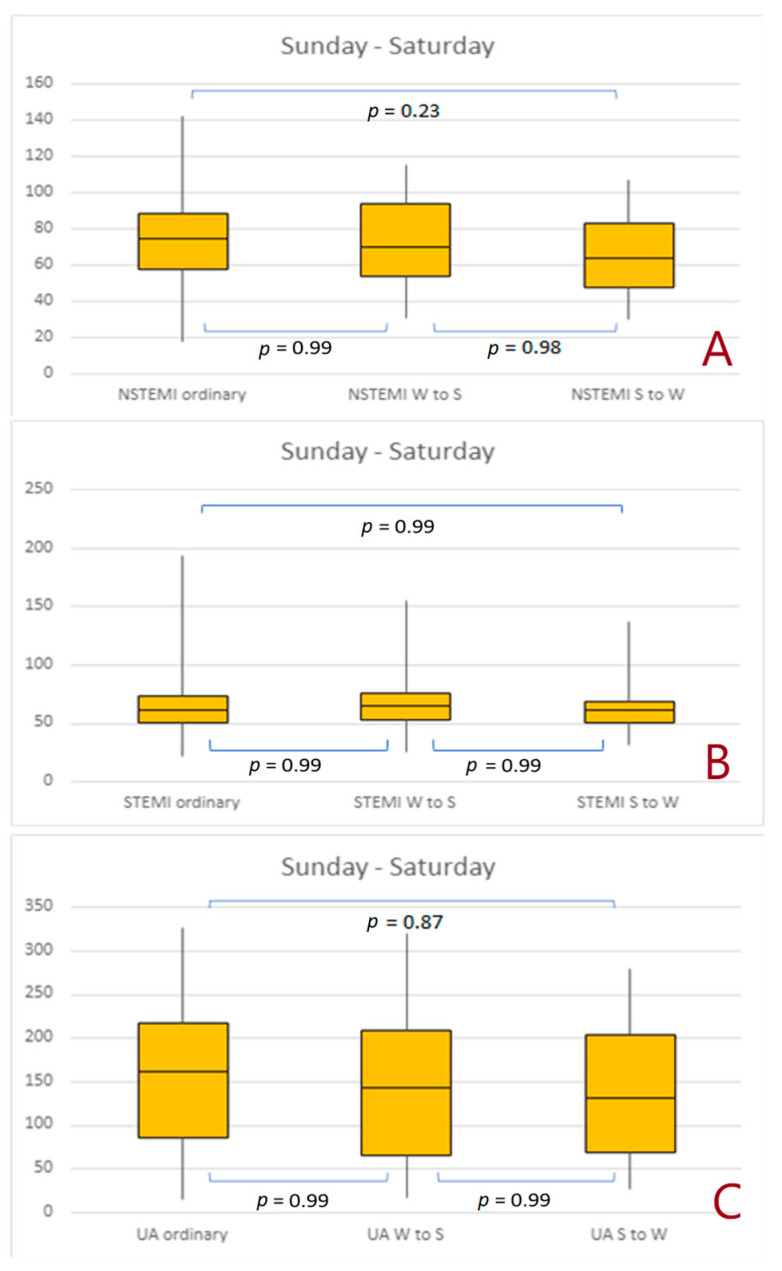
Number of non-ST-elevation myocardial infarctions, ST-elevation myocardial infarctions, and unstable angina cases that occurred between Sunday and Saturday after the time change. (**A**) 1 day effect; (**B**) 3 days effect, (**C**) 7 days effect. Abbreviations: NSTEMI, non-ST-elevation myocardial infarction; STEMI, ST-elevation myocardial infarction; S to W, Summer to Winter; UA, unstable angina; W to S, Winter to Summer.

There were no significant differences in the number of NSTEMIs, STEMIs, and UA cases within 3 days of any change in time (Figure 4A–C) as well as within 7 days of any change in time (Figure 5A–C).

In the case of the analysis of all ACS, the autumn time change statistically significantly reduced the number of all types of ACS, both in the analysis from the 1st day to 3 days (RR 0.89 [0.81–0.98]; *p* = 0.02) and in the analysis from the 1st day to 7 days (RR 0.88 [0.83–0.93]; *p* = 0.0001). In the analysis of the impact of time change on the occurrence of STEMI, no statistically significant seasonal differences were found. When analyzing the influence of time change on the occurrence of NSTEMI, a statistically significant reduction in the number of invasive procedures was found regarding autumn time change in the analysis from the 1st day to 7 days (RR 0.88 [0.77–1.0]; *p* = 0.048).

When analyzing the effect of time change on the occurrence of unstable angina pectoris, a statistically significant reduction in the number of procedures was found regarding autumn time change in the analysis from the 1st day to 7 days (RR 0.87 [0.79–0.95]; *p* = 0.001). When analyzing both changes in time in UA, a statistically significant reduction in the number of treatments was found in the analysis from the 1st day to 7 days (RR 0.91 [0.83–0.99]; *p* = 0.03) (Table 1).

Poisson regression showed the influence of many clinical factors (age, weight, diabetes, history of myocardial infarction, previous CABG, active smoking status, psoriasis, hypertension, kidney disease, and COPD) on the occurrence of any ACS after autumn switching time (during, subsequently, 1 day and 3 days) and after both time changes within 7 days (Table S1). In our case, the Poisson multivariate analysis presents the influence of numerous factors and variables on the occurrence of ACS, NSTEMI, STEMI, and UA during the time change. The strongest predictors of ACS were previous CABG and hypertension.

Among the clinical factors, the occurrence of NSTEMI within 7 days from the time change was influenced by body weight, diabetes, previous myocardial infarction, prior PCI, active smoking status, hypertension, and COPD (Table S2). The occurrence of UA within 7 days after the autumn time change and after both time changes was influenced by body weight, previous myocardial infarction, previous CABG, active smoking status, psoriasis, arterial hypertension, kidney disease, and COPD (Table S3).

The analysis of the impact of time changes on the periprocedural mortality of patients with any ACS revealed the increase in the risk of death within 1 day of both changes in time, but also in the case of the autumn time change and spring time change. Perioprocedural mortality is considered as death 24 h after procedures. The increase in the risk of death in the period of 3 and 7 days from the time change was observed in the case of the spring time change and both time changes. In the case of STEMI, the spring time change and both time changes were associated with an increase in the risk of death within a 7-day perspective. In the case of NSTEMI, the autumn time change and both changes in time were associated with an increased risk of death. The change in time did not affect the risk of death in UA (Table 2).

## 4. Discussion

The disruption of biological rhythms may be associated with the occurrence of specific diseases. Changes of time in spring and autumn have been found to cause such disturbances.

Our analysis of data from the Polish national registry comprising patients who underwent PCI demonstrated no statistically significant seasonal differences associated with the change in time and the occurrence of STEMI. In contrast, when analyzing the impact of changes in DST on the occurrence of NSTEMI or UA, we noticed a significant reduction in the number of admissions regarding autumn change time.

The impact of changes in time on the incidence of ACS has been extensively studied. Based on a study of a group of 2412 patients with STEMI, Čulić V. et al. reported a higher incidence of STEMI occurrence in the first 4 business days after the spring DST switch, particularly on Monday [10].

Also, the autumn time change was characterized by a higher incidence rate of STEMI with peaks on Tuesday and Thursday [10]. Similar studies based on day-to-day analyses have already been conducted, but their statistics did not distinguish between types of heart attacks. Due to the size of our cohort, we could afford to do so. In studies conducted by Sipilä J. et al. in the years 2001–2009, they performed the analysis of the incidence of myocardial infarction and in-hospital mortality associated with DST in 6298 patients in Finland. The incidence of MI during the entire week of the study after DST was similar to that of the control weeks, both during the change to daylight-saving time and winter time. The incidence of STEMI increased on Wednesday after the spring transition, while after the autumn transition, it decreased on Monday, but increased on Thursday [11]. Our analysis conducted on a large group of patients from the ORPKI registry throughout Poland (874,031 PCI procedures) suggests that the autumn time change was significantly associated with fewer ACS PCI procedures irrespective of whether time intervals were analyzed from the first day to 3 days or from the first day to 7 days.

Previous studies when reporting the incidence of AMI did not specify whether there was a differential effect on STEMI and NSTEMI. Manfredini R. et al. in a meta-analysis of six large studies including 87,994 patients with a heart attack, performed between 2009 and 2016, reported a higher incidence of a heart attack during the spring change than the autumn change. A link with the occurrence of a heart attack and daylight-saving time has been confirmed [12]. However, a large analysis conducted in a group of 80,970 patients with ACS between 2015 and 2018 by Derks et al. showed no correlation between the time change and the occurrence of STEMI [7].

Several external factors are known to influence the occurrence of STEMI; for example, Čulić V. et al., who analyzed data of 10,519 patients with STEMI, reported that factors such as severe physical activity, moderate physical activity, eating a meal, various types of emotional stress (especially anger), meteorological stress, and sexual activity had a significant impact on the occurrence of STEMI at a given time [13].

The mortality of patients with STEMI is also influenced by sleep disorders. The switch from and into daylight-saving time can influence the sleep cycle. Medina D. et al. studied the effects of time change on the sleep and alertness of middle-school students. Sleep duration decreased by an average of 32 min per night of the week after DST, reflecting a cumulative sleep loss of 2 h 42 min compared to the baseline week (*p* = 0.001). Moreover, prolonged response time (*p* < 0.001) and increased daytime sleepiness (*p* < 0.001) were also demonstrated [14]. In hypertensive patients, the lack of sleep can increase sympathetic nervous system activity, leading to the increase in blood pressure and heart rate, and subsequently to a higher risk of cardiovascular events [15]. Sleep disorders (impaired awakening) were found to significantly affect the mortality after acute myocardial infarction (AMI) in males in a study conducted by Clark A. et al. In turn, in females included in the Swedish National Registry, sleep impairment translated into a higher risk of subsequent AMI, stroke, and heart failure, whereas no clear effects were found for case fatality. However, there was no effect on mortality [16]. Similar observations were reported by Jiddou M.R. et al. In a study conducted between October 2006 and April 2012 on a population of 935 patients (59% men and 41% women), time changes significantly affected the incidence of STEMI [17].

As a result of sudden changes in the circadian rhythm and sleep time limitations, there is also a change in the concentration of CRP, as demonstrated by Meier-Ewert H.K. et al. [18]. Healthy volunteers who had significant sleep time restrictions, both long-term and short-term, displayed a significant increase in blood CRP, which is a predictor of cardiovascular morbidity. The immediate visible effect of sleep deprivation is the stimulation of the sympathetic nervous system, resulting in an increase in blood pressure, an elevated heart rate, and increased flow through the coronary arteries. These arteries, by twisting and bending, make the atherosclerotic plaque less stable and simultaneously activate blood platelets. Intensified inflammatory processes additionally increase the thrombogenic potential of the atherosclerotic plaque, facilitating the formation of blood clots [19]. These events, in combination with chronic factors such as long-term sleep deprivation, the disruption of the biological clock, and the presence of atherosclerotic plaques, can initiate a heart attack within a few days of a time change [20]. Valdez et al. summarized studies on the transition to daylight-saving time, where it was observed that individuals who do not easily adapt to the time change experience difficulties falling asleep, daytime sleepiness, and fatigue, which contribute to higher stress levels, a well-known factor in heart attacks [21]. In an analysis by Harrison et al., the results of studies were summarized, indicating that time changes affect the quality and duration of sleep, and these disturbances persist for about a week. Initially, the impact of these changes on the body was underestimated, but with an increasing number of analyses, behavioral disturbances, increased stress, and reduced concentration levels were observed, leading to a higher incidence of road accidents [22].

The use of a logistic regression analysis using the Poisson method allows for a well-conducted determination of the impact of numerous factors analyzed in our study on periprocedural death during a time change. Multivariate analysis methods are a good tool for such analyses [23].

Our research also has its limitations. We analyzed only data obtained from these ACS patients who underwent interventional cardiology procedures. We do not know the actual number of all ACS admissions after DST, since a proportion of NSTEMI and UA particularly is pharmacologically managed, so we did not capture such data in our PCI registry.

## 5. Conclusions

1. The analysis of the differences in the number of ACS from the first day to 7 days revealed that the number of ACS was lower for both changes over time; moreover, the autumn time change statistically significantly reduced the number of treatments, also in the analysis from the first day to 3 days.
2. In the analysis of the influence of both time changes on the occurrence of STEMI, no statistically significant seasonal differences were found.
3. The autumn time change is associated with a less frequent occurrence of NSTEMI within 7 days.
4. A change in time may contribute to the increase in periprocedural mortality in ACS (STEMI and NSTEMI).

## Figures and Tables

**Table 1:** The impact of time changes on occurrence of ACS.

	Sunday (1 Day)		Sunday–Tuesday (3 Days)		Sunday–Saturday (7 Days)	
	Rate Ratio (95% CI)	*p*-Value	Rate Ratio (95% CI)	*p*-Value	Rate Ratio (95% CI)	*p*-Value
ACS						
Winter to Summer	0.91 (0.72–1.14)	0.45	0.95 (0.86–1.05)	0.30	0.98 (0.92–1.04)	0.45
Summer to Winter	0.96 (0.75–1.20)	0.73	0.89 (0.81–0.98)	0.02	0.88 (0.83–0.93)	<0.001
Any	0.94 (0.74–1.18)	0.60	0.92 (0.83–1.01)	0.09	0.93 (0.87–0.99)	0.02
STEMI						
Winter to Summer	0.94 (0.63–1.42)	0.84	1.01 (0.83–1.24)	0.92	1.03 (0.91–1.17)	0.65
Summer to Winter	1.02 (0.68–1.52)	1	0.94 (0.77–1.15)	0.55	0.93 (0.81–1.06)	0.28
Any	0.98 (0.66–1.47)	0.98	0.98 (0.80–1.19)	0.84	0.98 (0.86–1.11)	0.77
NSTEMI						
Winter to Summer	0.88 (0.57–1.35)	0.60	0.97 (0.80–1.17)	0.77	0.97 (0.86–1.1)	0.71
Summer to Winter	0.96 (0.63–1.46)	0.91	0.86 (0.71–1.05)	1.51	0.88 (0.77–1.0)	0.048
Any	0.92 (0.60–1.41)	0.76	0.92 (0.75–1.12)	0.41	0.93 (0.82–1.05)	0.23
UA						
Winter to Summer	0.94 (0.63–1.41)	0.85	0.91 (0.79–1.04)	0.20	0.95 (0.87–1.04)	0.26
Summer to Winter	0.93 (0.62–1.39)	0.93	0.89 (0.77–1.02)	0.09	0.87 (0.79–0.95)	0.001
Any	0.93 (0.62–1.39)	0.77	0.90 (0.78–1.03)	0.12	0.91 (0.83–0.99)	0.03

Abbreviations: ACS, acute coronary syndrome; NSTEMI, non-ST-elevation myocardial infarction; STEMI, ST-elevation myocardial infarction; UA, unstable angina.

**Table 2:** The impact of time change on mortality in ACS.

	Sunday (1 Day)		Sunday–Tuesday (3 Days)		Sunday–Saturday (7 Days)	
	Odds Ratio (95% CI)	*p*-Value	Odds Ratio (95% CI)	*p*-Value	Odds Ratio (95% CI)	*p*-Value
ACS						
Winter to Summer	2.58 (1.42–1.67)	0.002	1.61 (1.17–2.21)	0.003	1.36 (1.1–1.7)	0.006
Summer to Winter	2.01 (1.04–3.88)	0.037	1.22 (0.84–1.78)	0.29	1.13 (0.88–1.46)	0.32
Any	2.29 (1.47–3.56)	0.0002	1.43 (1.12–1.82)	0.004	1.26 (1.07–1.49)	0.007
STEMI						
Winter to Summer	1.9 (0.94–3.84)	0.07	1.41 (0.95–2.1)	0.09	1.42 (1.1–1.85)	0.008
Summer to Winter	0.87 (3.2–2.33)	0.78	1.09 (0.68–1.74)	0.71	1.2 (0.89–1.61)	0.24
Any	1.36 (0.77–2.42)	0.29	1.26 (0.93–1.7)	0.14	1.32 (1.08–1.61)	0.006
NSTEMI						
Winter to Summer	1.52 (0.38–6.12)	0.55	1.38 (0.72–2.67)	0.34	0.95 (0.57–1.58)	0.84
Summer to Winter	3.51 (1.45–8.5)	0.005	1.37 (0.68–2.76)	0.37	0.84 (0.47–1.48)	0.55
Any	2.56 (1.21–5.41)	0.01	1.38 (0.85–2.24)	0.19	0.89 (0.61–1.31)	0.57
UA						
Winter to Summer	3.59 (0.5–25.61)	0.2	2.34 (0.97–5.68)	0.06	1.59 (0.82–3.09)	0.17
Summer to Winter	0.0001 (0.0000–1.18 × 10^137^)	0.96	0.95 (0.24–3.84)	0.95	1.15 (0.51–2.59)	0.73
Any	1.82 (0.25–12.95)	0.55	1.66 (0.79–3.52)	0.18	1.39 (0.83–2.34)	0.21

Abbreviations: ACS, acute coronary syndrome; NSTEMI, non-ST-elevation myocardial infarction; STEMI, ST-elevation myocardial infarction; UA, unstable angina.

## Data Availability

The datasets generated during and/or analyzed during the current study are available from the corresponding author upon reasonable request.

## Supplementary Materials

The following supporting information can be downloaded at: https://www.mdpi.com/article/10.3390/jcdd10090375/s1, Table S1: Factors affecting the occurrence of ACS. CABG—coronary artery bypass graft; COPD—Chronic obstructive pulmonary disease; IRR—incidence rate ratio; MI—myocardial infarction; PCI—percutaneous coronary intervention. Table S2: Factors affecting the occurrence of NSTEMI. CABG—coronary artery bypass graft; COPD—Chronic obstructive pulmonary disease; IRR—incidence rate ratio; MI—myocardial infarction; PCI—percutaneous coronary intervention. Table S3: Factors affecting the occurrence of UA. CABG—coronary artery bypass graft; COPD—Chronic obstructive pulmonary disease; IRR—incidence rate ratio; MI—myocardial infarction; PCI—percutaneous coronary intervention.
