# Supplementary material for: The Effect of Daylight-Saving Time on Percutaneous Coronary Intervention Outcomes in Acute Coronary Syndrome Patients—Data from the Polish National Registry of Percutaneous Coronary Interventions (ORPKI) in the Years 2014–2022
Source: J Cardiovasc Dev Dis. 2023 Sep 1;10(9):375. doi: 10.3390/jcdd10090375 (PMC10532136; doi:10.3390/jcdd10090375)
Supplement: Supplementary file 1 [file jcdd-10-00375-s001.zip › jcdd-2519376-supplementary.pdf]

Table S1. Factors affecting the occurrence of ACS.

Variable	Sunday – Tuesday (3 days) Summer to Winter		Sunday – Saturday (7 days) Summer to Winter		Sunday – Saturday (7 days) Any	
	IRR (95% CI)	P value	IRR (95% CI)	P value	IRR (95% CI)	P value
Age, years	0.98 (0.97 – 0.99)	< 0.001	0.98 (0.97 – 0.99)	< 0.001	0.98 (0.97 – 0.99)	< 0.001
Gender (male)	1.03 (0.81 – 1.31)	0.8	1.04 (0.82 – 1.32)	0.76	1.04 (0.82 – 1.32)	0.76
Weight, kg	0.95 (0.94 – 0.95)	< 0.001	0.95 (0.94 – 0.95)	< 0.001	0.95 (0.94 – 0.95)	< 0.001
Diabetes	1.53 (1.14 – 2.04)	< 0.001	1.52 (1.14 – 2.03)	0.004	1.52 (1.14 – 2.03)	0.004
Previous Stroke	1.07 (0.51 – 2.25)	0.87	1.05 (0.5 – 2.22)	0.9	1.05 (0.5 – 2.22)	0.9
Previous MI	0.71 (0.52 – 0.97)	0.03	0.71 (0.52 – 0.97)	0.03	0.71 (0.52 – 0.97)	0.03
Previous PCI	0.75 (0.54 – 1.04)	0.08	0.75 (0.54 – 1.03)	0.08	0.75 (0.54 – 1.03)	0.08
Previous CABG	9.94 (6.0 – 16.5)	< 0.001	9.87 (5.97 – 16.34)	< 0.001	9.87 (5.97 – 16.34)	< 0.001
Smoking status	0.1 (0.07 – 0.13)	< 0.001	0.1 (0.07 – 0.13)	< 0.001	0.1 (0.07 – 0.13)	< 0.001
Psoriasis	0.09 (0.1 – 0.63)	0.02	0.09 (0.01 – 0.63)	0.015	0.09 (0.01 – 0.63)	0.02
Hypertension	4.75 (3.77 – 5.99)	< 0.001	4.75 (3.77 – 5.98)	< 0.001	4.75 (3.77 – 5.98)	< 0.001
Kidney disease	0.28 (0.16 – 0.48)	< 0.001	0.28 (0.16 – 0.48)	< 0.001	0.28 (0.16 – 0.48)	< 0.001
COPD	0.08 (0.04 – 0.14)	< 0.001	0.08 (0.04 – 0.14)	< 0.001	0.08 (0.04 – 0.14)	< 0.001

Abbreviations: ACS, acute coronary syndrome; CABG, coronary artery bypass grafting; COPD, chronic obstructive pulmonary disease; IRR, incidence rate ratio; MI, myocardial infarction; PCI, percutaneous coronary intervention.

Table S2. Factors affecting the occurrence of NSTEMI.

Variable	Sunday – Saturday (7 days) Summer to Winter	
	IRR (95% CI)	P value
Age, years	0.9985 (0.99 – 1.00)	0.62
Gender (male)	1.08 (0.95 – 1.23)	0.25
Weight, kg	0.98 (0.98 – 0.98)	< 0.001
Diabetes	1.23 (1.06 – 1.43)	0.007
Previous Stroke	1.08 (0.8 – 1.46)	0.61
Previous MI	1.36 (1.14 – 1.62)	0.001
Previous PCI	0.45 (0.39 – 0.53)	< 0.001
Previous CABG	1.12 (0.85 – 1.47)	0.42
Smoking status	0.74 (0.64 – 0.85)	< 0.001
Psoriasis	0.63 (0.24 – 1.65)	0.34
Hypertension	1.39 (1.22 – 1.58)	< 0.001
Kidney disease	0.91 (0.73 – 1.14)	0.43
COPD	0.23 (0.17 – 0.31)	< 0.001

Abbreviations: ACS, acute coronary syndrome; CABG, coronary artery bypass grafting; COPD, chronic obstructive pulmonary disease; IRR, incidence rate ratio; MI, myocardial infarction; PCI, percutaneous coronary intervention.

Table S3. Factors affecting the occurrence of UA.

Variable	Sunday – Saturday (7 days) Summer to Winter		Sunday – Saturday (7 days) Any	
	IRR (95% CI)	P value	IRR (95% CI)	P value
Age, years	0.99 (0.98 – 1.01)	0.28	0.99 (0.98 – 1.0)	0.29
Gender (male)	1.09 (0.82 – 1.45)	0.54	1.1 (0.83 – 1.46)	0.5
Weight, kg	0.97 (0.96 – 0.97)	< 0.001	0.96 (0.96 – 0.97)	< 0.001
Diabetes	1.08 (0.78 – 1.5)	0.66	1.07 (0.77 – 1.49)	0.67
Previous Stroke	2.11 (0.89 – 4.99)	0.09	2.06 (0.87 – 4.86)	0.1
Previous MI	0.3 (0.21 – 0.43)	< 0.001	0.3 (0.21 – 0.43)	< 0.001
Previous PCI	0.79 (0.57 – 1.08)	0.14	0.78 (0.56 – 1.07)	0.12
Previous CABG	5.3 (3.04 – 9.23)	< 0.001	5.29 (3.03 – 9.22)	< 0.001
Smoking status	0.29 (0.21 – 0.41)	< 0.001	0.29 (0.21 – 0.41)	< 0.001
Psoriasis	0.1 (0.01 – 0.97)	0.047	0.1 (0.01 – 0.98)	0.048
Hypertension	3.46 (2.68 – 4.46)	< 0.001	3.47 (2.69 – 4.48)	< 0.001
Kidney disease	0.4 (0.21 – 0.74)	0.004	0.4 (0.22 – 0.74)	0.004
COPD	0.25 (0.12 – 0.53)	< 0.001	0.25 (0.12 – 0.54)	< 0.001

Abbreviations: ACS, acute coronary syndrome; CABG, coronary artery bypass grafting; COPD, chronic obstructive pulmonary disease; IRR, incidence rate ratio; MI, myocardial infarction; PCI, percutaneous coronary intervention.
